# GLP-1 receptor agonists vs. SGLT-2 inhibitors: the gap seems to be leveling off

**DOI:** 10.1186/s12933-021-01400-9

**Published:** 2021-10-12

**Authors:** Dario Giugliano, Lorenzo Scappaticcio, Miriam Longo, Giuseppe Bellastella, Katherine Esposito

**Affiliations:** 1 Division of Endocrinology and Metabolic Diseases, Department of Advanced Medical and Surgical Sciences, University of Campania Luigi Vanvitelli, Naples, Italy; 2 Ph.D. of Translational Medicine, Chair of Endocrinology and Metabolic Diseases, Department of Advanced Medical and Surgical Sciences, University of Campania Luigi Vanvitelli, Naples, Italy; 3 Diabetes Unit, Department of Advanced Medical and Surgical Sciences, University of Campania Luigi Vanvitelli, Naples, Italy

**Keywords:** Type 2 diabetes, SGLT-2 inhibitors, GLP-1 receptor agonists, Cardiorenal benefits

## Abstract

Cardiovascular disease (CVD) remains the leading cause of death in patients with type 2 diabetes (T2D). Older age, prior heart failure (HF) and CV events, peripheral artery disease, and kidney complications can identify a subgroup of patients with T2D at high risk of mortality who are likely to achieve the greatest benefit from newer glucose-lowering agents. Both glucagon-like peptide-1 receptor agonists (GLP-1RA) and sodium-glucose cotransporter-2 (SGLT-2) inhibitors can reduce CV risk in patients with T2D, and both are recommended by the American Diabetes Association to reduce the risk of major cardiovascular events (MACE). The magnitude of the benefits of GLP-1RA and SGLT-2 inhibitors on MACE are similar, ranging from 12 to 14% reduction of risk, but only GLP-1RA may reduce the risk of stroke. The most striking difference between the two classes of drugs relates to the amelioration on hospitalization for HF, as the benefit of SGLT-2 inhibitors surpass by threefold that obtained with GLP-1RA. Despite this, GLP-1RA also exert a significant benefit on HF which suggest their use when SGLT-2 inhibitors are contraindicated or not tolerated. The difference between the two classes is less impressive for the kidney outcome. Overall, the results of CVOTs published so far seems to suggest that the gap between the cardiorenal benefits of SGLT-2 and GLP-1RA is narrowing.

In 1999, the American Heart Association stated that “diabetes is a cardiovascular disease” [1]. A review of 2018 including data from 57 articles involving 4 million people indicated that the overall prevalence of cardiovascular disease (CVD) in diabetic patients was 32.2% [2]. Still today, individuals with diabetes have an approximately two-fold increased risk of all-cause mortality than those without diabetes [3]. On the other hand, the cardiovascular destiny of the diabetic patient is not unavoidable, as patients with type 2 diabetes (T2D) who had major risk factors for cardiovascular disease (CVD) within the target range had little or no excess risk of CVD and mortality [4]. Unfortunately, only 5–6% of people with T2D had optimal risk factor control [4, 5]. In a contemporary cohort of 16,492 patients with T2D and at high/very high CV risk participating in the SAVOR-TIMI 53 trial [6], CVD remained the leading cause of death and approximately one-third of all deaths were classified as sudden. Older age, prior heart failure (HF) and CV events, peripheral artery disease, and kidney complications can identify a subgroup of patients with T2D at high risk of mortality who are likely to achieve the greatest benefit from aggressive management of modifiable risk factors and newer glucose-lowering agents.

Two classes of newer anti-hyperglycemic agents can reduce CV risk and events in patients with T2D, namely glucagon-like peptide-1 receptor agonists (GLP-1RA) and sodium-glucose cotransporter-2 (SGLT-2) inhibitors. GLP-1RA can reduce MACE (major cardiovascular events) and its individual components, CV death, myocardial infarction (MI) and stroke. These beneficial effects of GLP-1RA on MACE are independent of many variable including the presence of established CV disease at baseline, the structural basis of GLP-1RA (exendin-4 based agonists vs. human GLP-1- based molecules), the daily or weekly administration of the agonist, baseline HbA1c, body weight, age (> 65 vs. ≤ 65 years), baseline eGFR (<60 vs. ≥60 ml/min per 1.73 m^2^) and duration of follow-up of the trial (<3 vs. ≥ 3 years) [7, 8]. Intuitively, therapy with GLP-1RA can be beneficial in patients with T2D and established CVD or at risk for CVD. Accordingly, the last recommendation of the American Diabetes Association (ADA, Standard of Care 2021) states that in patients with T2D and established CVD or multiple risk factors for CVD, a GLP-1RA with demonstrated cardiovascular benefit is recommended to reduce the risk of MACE [9].

SGLT-2 inhibitors also reduce the risk of atherosclerotic MACE in patients with T2D with or without established CVD [10, 11]. SGLT-2 inhibitors reduce the risk of hospitalization for HF and progression of kidney disease in patients with established CVD, multiple risk factors for CVD, or diabetic kidney disease. According to this evidence, ADA states that in patients with T2D and established CVD, multiple CVD risk factors, or diabetic kidney disease, an SGLT-2 inhibitor with demonstrated cardiovascular benefit is recommended to reduce the risk of MACE and/or hospitalization for HF [9]. Moreover, in patients with T2D and established HF with reduced ejection fraction (HFrEF), an SGLT-2 inhibitor with proven benefit in this patient population is recommended to reduce the risk of worsening HF and CV death. The benefits seen in this patient population likely represent a class effect, and they appear unrelated to glucose lowering given comparable outcomes in HFrEF patients with and without diabetes. For many patients, use of either an SGLT-2 inhibitor or a GLP-1RA to reduce CV risk is appropriate. It is unknown whether use of both classes of drugs will provide an additive cardiovascular benefit.

Ongoing trials are assessing the effects of several SGLT-2 inhibitors in patients with HF and reduced or preserved ejection fraction. The results of the EMPEROR-P [12] have shown that in 5988 patients with or without T2D and with HF and preserved ejection fraction (>40%), treatment with 10 mg empagliflozin for a median time of 26.2 months reduced the risk of a composite of CV death or hospitalization for HF by 21%, and effect which was mainly related to a 29% lower risk of hospitalization for HF. The benefit of empagliflozin was independent of the diabetic status. According to the results of both DAPA-HF [13] and EMPEROR-R [14], the FDA have approved both dapagliflozin and empagliflozin to reduce the risk of CV death or hospitalization for HF in adults with HF and reduced ejection fraction regardless of whether they have diabetes.

The magnitude of the benefits of GLP-1RA and SGLT-2 inhibitors on MACE are similar in patients with T2D, ranging from 12 to 14% reduction of risk, but only GLP-1RA may reduce the risk of stroke (Fig. 1). The most striking difference between the two classes of drugs relates to the amelioration on hospitalization for HF, as the benefit of SGLT-2 inhibitors surpass by threefold that obtained with GLP-1RA. Despite this, GLP-1RA also exert a significant benefit on HF which suggests their use when SGLT-2 inhibitors are contraindicated or not tolerated. The difference between the two classes is less impressive for the kidney outcome; moreover, similar GLP-1RA effect sizes suggest a lack of sufficient power rather than a lack of effect. Overall, the results of CVOTs published so far seems to suggest that the gap between the cardiorenal benefits of SGLT-2 and GLP-1RA is narrowing.

**Fig. 1:**
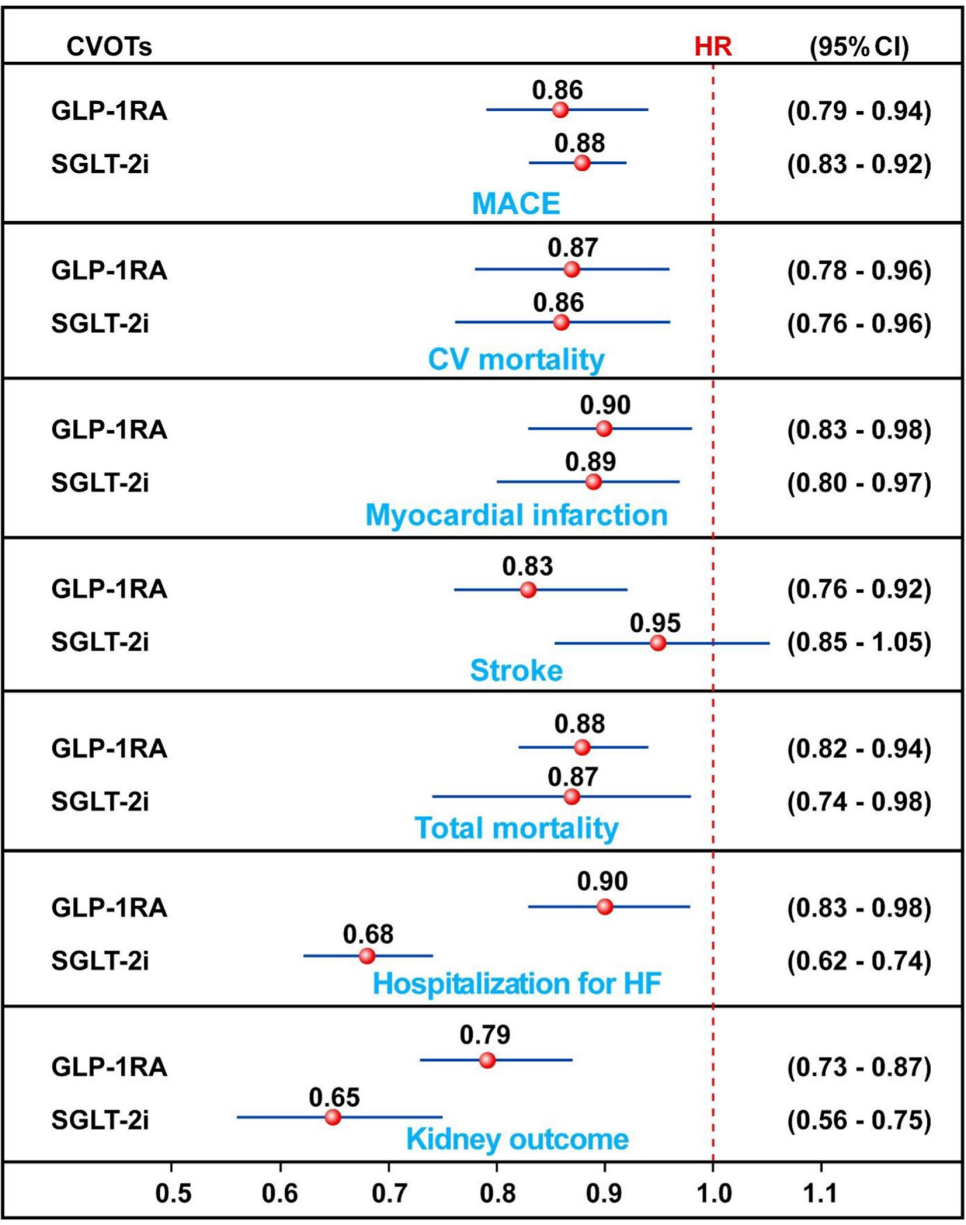
Meta-analyses of cardiorenal effects exerted by GLP-1RA and SGLT-2 inhibitors in patients with or without type 2 diabetes. *HR* hazard ratio, *CI* confidence intervals. The results are based on data in Ref. [7, 8, 11]

Previous analyses have suggested a larger benefit of SGLT-2 inhibitors, as compared with GLP-1RA, on cardiorenal events. For instance, a large network meta-analysis showed that SGLT-2 inhibitors reduced hospitalization for HF and renal composite outcome more than GLP-1RA [15], and that in patients with cardiovascular disease, SGLT-2 inhibitors show significant reduction in both heart failure (HF) hospitalizations and mortality for patients with HF and reduced ejection fraction [16].

Moreover, a retrospective real-world study shows that both GLP1-RA and SGLT-2 inhibitors reduce the 10-year risk for cardiovascular disease in patients with T2D in primary cardiovascular prevention [17], although SGLT-2 inhibitors seem to have a greater cardioprotective benefit compared to GLP-1RA when used for secondary prevention among adults with T2D [18].

Prescriptions of the newer anti-hyperglycemic agents continue to stagnate, even among eligible patients [19], which may be related, at least in part, to the uncertainty about the optimal clinical management of T2D. Sources of uncertainty originate from the panoply of glycemic targets, the complexity of drug therapy, the choice of the first drug, the ideal sequence of drugs after the first drug failure, the possible harms of anti-hyperglycemic drugs, the outcomes of treatment (surrogate versus clinical) and the hierarchy of risk factors to treat for preventing the vascular complications. Ironically, multiple treatment guidelines and algorithms periodically released to improve guidance may generate confusion into clinicians [20]. Moreover, treatment algorithms cannot be truly evidence-based because of a lack of studies comparing all available treatment combination options. Confusion likely contributes to clinical inertia [20, 21], thereby effectively denying evidence-based treatments advocated to patients with T2D and CVD. Coordinated action is required to ensure that people with type 2 diabetes, cardiovascular disease, heart failure, or chronic kidney disease are treated appropriately with an SGLT-2 inhibitor or GLP-1RA. Moreover, more adults with diabetes in the US have suboptimal glycemic control now compared to 10 years ago [22]. In adult NHANES (National Health and Nutrition Examination Survey) participants with diagnosed diabetes, glycemic control declined after more than a decade of progress, associated with a resurgence in vascular diabetic complications [23].

SGLT-2 inhibitors and GLP-1RA represent antihyperglycemic therapies shown to reduce CVD and chronic kidney disease risks in patients with T2D. In addition, SGLT-2 inhibitors have shown benefit in patients with both HFrEF and HFpEF independently of diabetes status, which opens exciting possibilities for the use of these therapies in patients at risk for or with established CV or kidney disease without T2D. There is an urgent need to incorporate multidisciplinary care in the identification of high-risk patients who may benefit from these agents [24]. Finally, legislative support should promote equitable access to these agents, especially for vulnerable and underrepresented patient populations who also carry the highest burden of CVD and CKD risk with T2D.

## Data Availability

All data generated or analyzed during this study are included in this published article.

## Abbreviations

CVD: Cardiovascular disease
T2D: Type 2 diabetes
CV: Cardiovascular
HF: Heart failure
GLP-1RA: Glucagon-like peptide 1 receptor agonist
SGLT-2: Sodium-glucose cotransporter 2
MACE: Major cardiovascular events
MI: Myocardial infarction
ADA: American Diabetes Association
HFrEF: Heart failure with reduced ejection fraction
HFpEF: Heart failure with preserved ejection fraction
FDA: Food and Drug Administration
NHANES: National Health and Nutrition Examination Survey
CKD: Chronic kidney disease
